# Deep-Learning-Assisted Integrated Microfluidic Platform for Automated Analysis of EV RNA Mutations

**DOI:** 10.3390/mi17101193

**Published:** 2026-10-15

**Authors:** Yunxing Lu, Xiaohan Wang, Qingchun Bai, Qiang Shi, Yiman Song, Lin Zhou, Zhenhua Wu, Hongju Mao

**Affiliations:** 1 School of Science and Technology, Shanghai Open University, Shanghai 200433, China; 2 The International Peace Maternity and Child Health Hospital, School of Medicine, Shanghai Jiao Tong University, Shanghai 200030, China; 3 Shanghai Key Laboratory of Embryo Original Diseases, Shanghai 200030, China; 4 Shanghai Engineering Research Center of Open Distance Education, Shanghai Open University, Shanghai 200433, China; 5 State Key Laboratory of Transducer Technology, Shanghai Institute of Microsystem and Information Technology, Chinese Academy of Sciences, Shanghai 200050, China; 6 School of Stomatology, Dalian Medical University, Dalian 116044, China; 7 Center for Medical Research and Innovation, Shanghai Pudong Hospital, Fudan University Pudong Medical Center, Shanghai 200120, China

**Keywords:** extracellular vesicle, exosome, RNA mutation, droplet digital PCR, microfluidics, deep learning, liquid biopsy

## Abstract

Extracellular vesicles (EVs) carry key genetic biomarkers, such as somatic mutations, making them valuable targets for non-invasive cancer diagnostics. However, standard workflows that isolate EVs and detect low-abundance mutations typically require multi-step manual operations, leading to sample loss and potential cross-contamination. Here, we present an integrated microfluidic platform combined with a deep learning algorithm for the continuous processing and automated droplet analysis of EV-derived RNA mutations. The microfluidic chip integrates magnetic bead-based EV capture, online lysis, droplet generation, and arrayed RT-droplet digital PCR (RT-ddPCR) on a single device. To streamline signal readout, a deep learning framework based on Cellpose and K-means clustering was implemented to process individual fluorescence images, enabling droplet detection, background removal, gray-scale intensity extraction, and automatic positive/negative droplet classification. The system showed a linear response across a wide range of original-sample inputs (R^2^ = 0.9950). The platform was evaluated using EV samples derived from three cancer cell lines (NCI-H1975, SK-LU-1, and HepG2) and the detection of EGFR L858R and KRAS mutations were achieved. By coupling continuous sample processing with automated image interpretation, this platform offers a practical reference for integrated EV nucleic acid analysis for liquid-biopsy applications.

## 1. Introduction

Liquid biopsy is extending cancer diagnostics by enabling molecular information to be obtained from minimally invasive samples rather than tissue biopsies [1,2,3,4]. Extracellular vesicles (EVs) are membrane-bound vesicles secreted by cells into biological fluids, carrying proteins, lipids, and nucleic acids that reflect the status of parental cells [5,6,7,8,9]. In particular, EV-derived messenger RNA (mRNA) and microRNA (miRNA) serve as potential biomarkers for liquid biopsy in oncology. Detecting specific mutations in EV-derived RNA allows for non-invasive monitoring of tumor dynamics and treatment resistance [10,11,12,13,14].

Droplet digital PCR (ddPCR) provides absolute quantification and high sensitivity for low-abundance nucleic acid targets by partitioning the reaction mixture into thousands of picoliter-scale droplets [15,16,17,18]. Coupled with reverse transcription (RT-ddPCR), this approach is well-suited for analyzing low-copy EV RNA targets. However, conventional EV RT-ddPCR workflows typically involve distinct steps—EV isolation, RNA extraction, reaction assembly, droplet generation, thermal cycling, and signal readout—that are performed manually across multiple instruments [19,20,21]. These manual transfers increase hands-on time, introduce variability, and heighten the risk of sample contamination or loss [20,22,23,24].

Microfluidic technologies provide an advantageous approach to address these integration challenges by performing liquid handling steps on a single microchip. However, maintaining stable droplet generation and dense array retention following sample enrichment remains non-trivial. Furthermore, optical signal interpretation presents an additional bottleneck [25,26,27,28]. Deep learning algorithms, such as Convolutional Neural Networks (CNNs), U-Net variants, and Mask R-CNN, have recently been introduced to automate microfluidic and dPCR image analysis, demonstrating strong capabilities in object detection and semantic segmentation [29,30,31,32,33,34,35,36]. Despite these advances, many existing approaches still face challenges when processing raw fluorescence micrographs captured under non-ideal illumination, where background noise, packing edge effects, and local intensity fluctuations frequently necessitate manual threshold adjustment [37,38,39,40,41]. Developing an automated, stable algorithm capable of segmenting dense droplet arrays and categorizing fluorescence states from fluorescence images remains essential for practical microfluidic ddPCR readout.

Reverse-transcription droplet digital PCR is particularly suited to low-abundance mutation analysis because it partitions EV-derived RNA into thousands of independent reaction units, allowing for mutation-associated molecules to be resolved as discrete endpoint events rather than being averaged across the bulk reaction volume. Compared with RT-qPCR, this digital format reduces the masking effects of abundant non-mutated targets and bulk fluorescence background and enables calibration-free quantification and reproducible rare-variant measurements [42,43,44,45,46]. Multiplex fluorescent-probe strategies further allow for target and sequence-specific signals to be distinguished within partitioned reactions [47,48].

In this work, we present an integrated microfluidic platform designed for the streamlined processing of EV-derived RNA and automated signal quantification via deep learning. The chip sequentially performs magnetic bead-based EV capture, online lysis, droplet emulsification, and stable droplet retention within a unified microfluidic system. For signal reading, a deep learning framework based on Cellpose segmentation and K-means clustering was applied directly to raw fluorescence images, executing automated droplet identification, background subtraction, gray-scale intensity profiling, and binary classification without human threshold setting. The functionality of the platform was demonstrated on EVs derived from NCI-H1975, SK-LU-1, and HepG2 cell lines to resolve EGFR L858R and KRAS mutation profiles. The resulting workflow couples stable microfluidic operation with efficiency droplet-level readouts and provides a foundation for extending mutation analysis to broader EV RNA applications.

## 2. Materials and Methods

### 2.1. Materials and Reagents

Polydimethylsiloxane (PDMS, Sylgard 184) and the corresponding curing agent were obtained from Dow Corning (Midland, MI, USA). Triton X-100 and trichloro-(1H,1H,2H,2H-perfluorooctyl) silane (PFOTS) were purchased from Sigma-Aldrich (St. Louis, MO, USA). For microfluidic fabrication, PDMS prepolymer, curing agent, and Triton X-100 were mixed at a weight ratio of 10:1:0.05. Glass slides and 50 μm thick double-sided adhesive (DSA) were used to construct the droplet reaction chamber. Mineral oil (Thermo Fisher Scientific, Waltham, MA, USA) was used as the continuous phase for droplet generation.

Anti-CD63-functionalized magnetic beads and the corresponding EV lysis/binding reagents were obtained from Thermo Fisher Scientific (Waltham, MA, USA) and used for immunomagnetic EV capture and on-chip lysis. One-step RT-ddPCR reaction reagents were used for reverse transcription and digital amplification of EV-derived RNA. Primers and TaqMan probes targeting EGFR L858R and KRAS were synthesized by Integrated DNA Technologies (IDT, Coralville, IA, USA). FAM- and HEX-labeled probes were used to distinguish total-target and mutation-specific signals in the corresponding fluorescence channels. Phosphate-buffered saline (PBS; Sangon Biotech, Shanghai, China) and RNase-free water were used for sample dilution, washing, and preparation of nucleic-acid reaction solutions. All aqueous solutions for RNA processing were prepared using RNase-free water.

### 2.2. Cell Culture and EV Sample Preparation

Human non-small cell lung cancer cell lines NCI-H1975 and SK-LU-1 and the human hepatocellular carcinoma cell line HepG2 were obtained from the American Type Culture Collection (ATCC, Manassas, VA, USA). NCI-H1975 and SK-LU-1 cells were cultured in RPMI-1640 medium (Gibco, Thermo Fisher Scientific, Waltham, MA, USA), whereas HepG2 cells were maintained in Dulbecco’s modified Eagle medium (DMEM; Gibco, Thermo Fisher Scientific, Waltham, MA, USA). The culture media were supplemented with 10% (*v*/*v*) fetal bovine serum (FBS) and 1% penicillin–streptomycin (both from Gibco, Thermo Fisher Scientific, Waltham, MA, USA). All cells were maintained in a humidified incubator at 37 °C with 5% CO_2_ and routinely passaged before reaching complete confluence.

For EV collection, cells were cultured to approximately 80% confluence, washed with PBS, and subsequently maintained under serum-free conditions for EV collection. Conditioned medium was collected after 48 h and sequentially centrifuged at 2000× *g* for 30 min and 10,000× *g* for 30 min at 4 °C to remove residual cells, cellular debris, and larger vesicular components. The resulting supernatant was carefully collected for subsequent EV preparation and analysis.

The particle concentration and size distribution of the isolated EV samples were characterized by nanoparticle tracking analysis (NTA). For NTA measurements, 10 μL of EV sample was diluted in PBS to an appropriate particle concentration of approximately 10^8^–10^9^ particles/mL. For TEM characterization, EVs were isolated from the pre-cleared cell-culture supernatants by ultracentrifugation at 100,000× *g* for 70 min at 4 °C. The ultracentrifugation step was repeated once to improve sample purity, and the resulting EV pellet was resuspended in sterile PBS.

For integrated microfluidic analysis, the EV-containing samples were introduced into the on-chip processing chamber and incubated with anti-CD63-functionalized magnetic beads. Following immunomagnetic capture, an external magnet was applied beneath the processing chamber to retain the EV-bound beads, and the supernatant was removed. Lysis buffer was subsequently introduced to release EV-associated RNA, followed by addition of the RT-ddPCR reaction mixture for downstream droplet generation and digital mutation analysis.

### 2.3. Microfluidic Device Design and Fabrication

The microfluidic device consisted of a large-volume EV processing chamber, a transfer microtube, a cross-shaped droplet-generation structure, a venting region, and a glass reaction chamber for droplet retention and thermal amplification. The processing chamber was designed as a cylindrical through-hole with a diameter of 8 mm and a height of 5 mm, providing a sample-processing capacity greater than 120 μL. The downstream droplet-generation structure was connected to the output of processing chamber through a microtube, allowing for the prepared reaction mixture to be transferred directly without pipetting. The droplet reaction chamber was constructed from two glass slides separated by a 50 μm thick double-sided adhesive layer, providing a shallow space for the formation and retention of a single-layer droplet array. A venting gap was incorporated between the microchannel and the reaction chamber to facilitate the release of air bubbles generated during fluid transfer or thermal cycling. The widths of the aqueous-phase inlet, continuous-phase inlet and downstream outlet channels were 150, 150 and 35 μm, respectively, and the channel height was 50 μm.

The patterned silicon mold was fabricated using photolithography and deep reactive ion etching. Briefly, the microfluidic pattern was designed using AutoCAD 2018 software and transferred to a chromium photomask, followed by photolithographic patterning on a silicon wafer. The patterned wafer was subsequently etched by deep reactive ion etching and cleaned with sulfuric acid. The silicon mold was treated overnight with trichloro-(1H,1H,2H,2H-perfluorooctyl) silane vapor to facilitate subsequent PDMS release.

For device fabrication, PDMS prepolymer, curing agent, and Triton X-100 were mixed at a weight ratio of 10:1:0.05 and degassed under vacuum. The mixture was poured onto the silicon mold and cured at 80 °C for 50 min. After curing, the PDMS replica was peeled from the mold, and the inlet and outlet ports were punched. The patterned PDMS layer was then bonded to a clean glass substrate following air-plasma treatment. A microtube was inserted to connect the outlet of the processing chamber with the aqueous inlet of the droplet generator. The reaction chamber was assembled using two glass slides separated by a 50 μm thick double-sided adhesive spacer.

### 2.4. Microfluidic On-Chip Operations

The integrated on-chip workflow included immunomagnetic EV capture, EV lysis, reaction-mixture preparation, droplet generation, and droplet loading into the downstream reaction chamber. EV samples were first introduced into the large-volume processing chamber and mixed with anti-CD63-functionalized magnetic beads. After sufficient incubation, an external magnet was positioned beneath the chamber to collect and retain the EV-bound magnetic beads, while the supernatant was removed. Lysis buffer was subsequently added to release EV-associated RNA, followed by addition of the RT-ddPCR reaction reagents to form the final aqueous reaction mixture. The overall processing sequence was performed within the chip without intermediate pipette transfer between EV isolation and reaction preparation. The immunomagnetic isolation and subsequent addition of lysis and reaction reagents followed the sequence shown for on-chip EV processing.

Approximately 25 μL of the prepared reaction mixture was transferred through the connecting microtube to the aqueous inlet of the cross-shaped droplet generator. The formulated mineral-oil continuous phase was introduced through the continuous-phase inlet. Both the aqueous reaction mixture and the continuous oil phase were pressure-driven at 100 mbar through their respective inlets, and the two phases converged at the flow-focusing junction to generate monodisperse droplets of approximately 50 μm in diameter. Up to approximately 100,000 droplets were generated in a single run and continuously transported into the glass reaction chamber, where they assembled into a densely packed single-layer array for subsequent RT-ddPCR thermal cycling.

### 2.5. Thermal Cycling and Fluorescence Imaging

After droplet generation and loading, the microfluidic device was transferred to a flat thermal-cycling platform for on-chip RT-ddPCR amplification. The droplets remained confined within the reaction chamber during thermal cycling, maintaining their dense arrangement for subsequent fluorescence imaging. After amplification, fluorescence images of the stationary droplet array were acquired using an inverted fluorescence microscope equipped with a high-sensitivity camera. FAM and HEX fluorescence channels were sequentially imaged using the corresponding excitation and emission filter sets. For each assay, total-target and mutation-specific fluorescence fields were acquired from the same stationary droplet region, allowing for signals from the two channels to be matched according to the spatial coordinates of individual droplets. Bright-field images were additionally acquired where required to evaluate droplet morphology and array stability after thermal cycling.

### 2.6. Deep Learning-Assisted Image Analysis and Signal Classification

A deep-learning-assisted image-processing pipeline was implemented for automated droplet segmentation, fluorescence quantification, and signal classification. The analysis workflow was developed in Python 3.9.23 using PyTorch 2.6.0 and OpenCV 4.11.0. Cellpose-based instance segmentation was performed using complementary model outputs generated from the source and enhanced fluorescence images. The cyto3 and nuclei models were used to identify candidate droplet masks, after which size filtering and mask merging were applied to retain non-duplicate droplet instances. For each detected droplet, a unique identifier and its centroid coordinates were recorded, together with the corresponding contour-derived area and equivalent radius. The analysis used pretrained Cellpose instance-segmentation weights together with inference settings tailored to densely packed microscopic droplet fields. Complementary source, locally enhanced and halo-suppressed representations were integrated with size filtering and mask merging to resolve adjacent droplets and retain contour-level boundaries and centroid coordinates rather than rectangular detection regions. The expected droplet diameter was set to 40 pixels and can be adjusted from 20 to 80 pixels, and droplets intersecting image boundaries were identified explicitly. Under this inference-based configuration, experimental micrographs are analyzed directly using a consistent parameter set; complete settings are provided in the Supplementary Methods S2 and S3. Higher displayed contrast facilitates direct visual inspection; however, the analytical workflow does not depend on display enhancement alone. Boundary recognition uses pixel-level spatial variation across the complementary image representations, whereas fluorescence is quantified from the original-resolution source image after local background correction. This design supports consistent analysis when droplet contrast is less pronounced or spatially non-uniform and reduces the manual workload and operator-dependent variation associated with visual counting.

Fluorescence measurements were performed on the original image rather than on the preprocessed images used for segmentation. For each droplet, the mean fluorescence intensity within an eroded inner region was calculated, while the mean intensity of a shape-matched surrounding ring was used to estimate the local background. The local background value was subtracted from the inner-droplet signal to reduce spatial illumination variations and background fluorescence. The resulting fluorescence contrast was subsequently used as the feature for unsupervised K-means clustering with k = 2. The cluster with the lower center was assigned as the negative population and that with the higher center as the positive population, allowing for automated positive/negative classification without manually defining a fluorescence threshold.

The analysis results for each image were exported at the individual-droplet level, including droplet identity, centroid coordinates, size-related parameters, fluorescence intensities, clustering results, and positive/negative classification. FAM and HEX images were analyzed separately using the same procedure.

## 3. Results

### 3.1. Integrated Workflow for EV RNA Mutation Analysis

**Figure 1:**
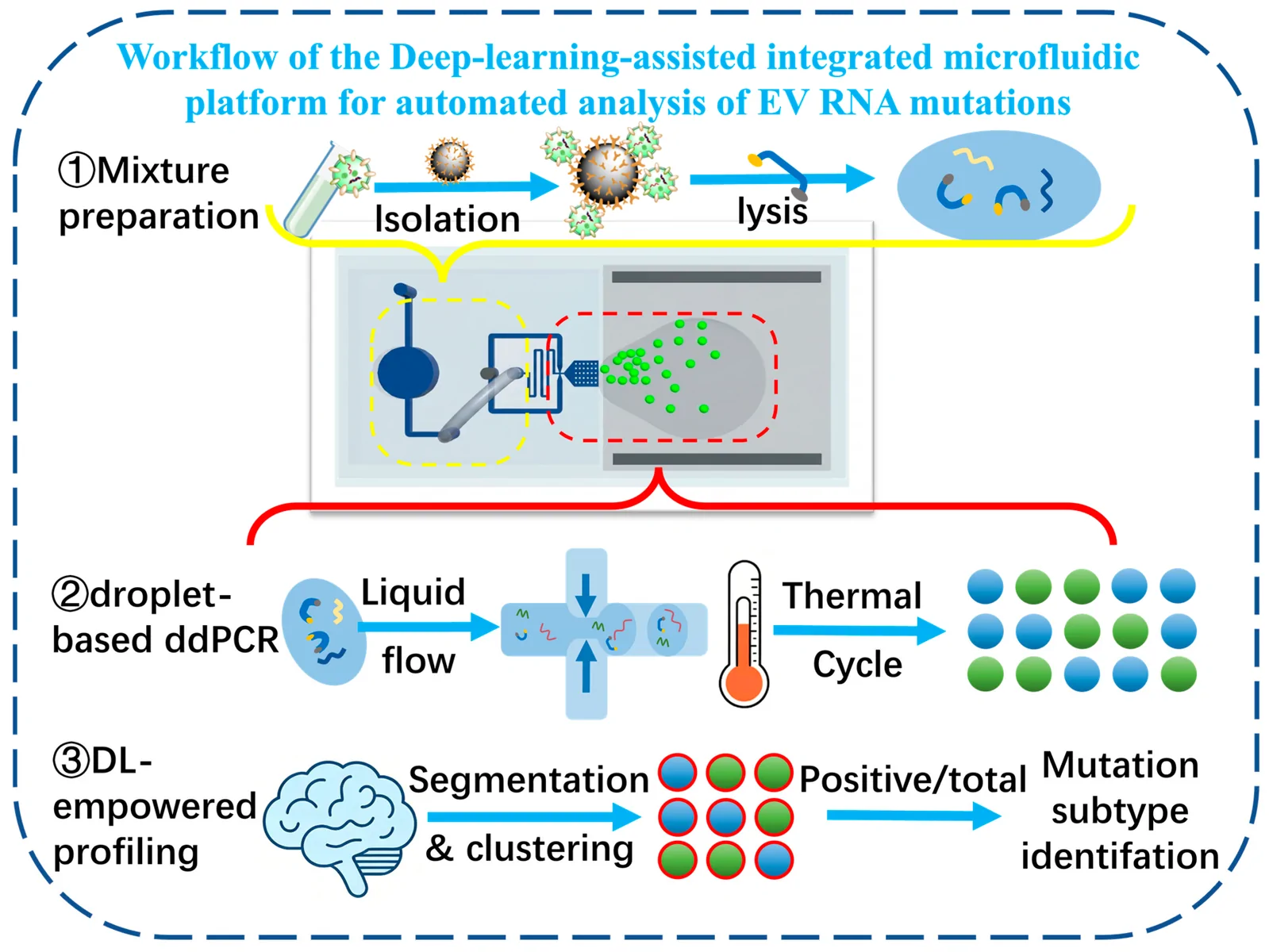
Workflow of the deep-learning-assisted integrated microfluidic platform for EV RNA mutation analysis. EVs are immunomagnetically captured and lysed on-chip, followed by mixture formation and RT-ddPCR droplet generation, thermal cycling, fluorescence imaging and model-assisted droplet analysis.

The integrated platform establishes an end-to-end continuous route from EV isolation to multiplexed target mutation fluorescence readout (Figure 1). Its large-volume processing chamber accommodates the initial sample and facilitates on-chip immunomagnetic capture, washing, lysis and reaction preparation before the mixture is seamlessly transferred to the downstream droplet generator and reaction chamber. Following thermal amplification, the resulting fluorescence fields are converted into droplet-resolved analytical outputs, linking the physical assay to automated interpretation within the same workflow.

### 3.2. Device Integration, Magnetic Capture and Droplet-Array Formation

**Figure 2:**
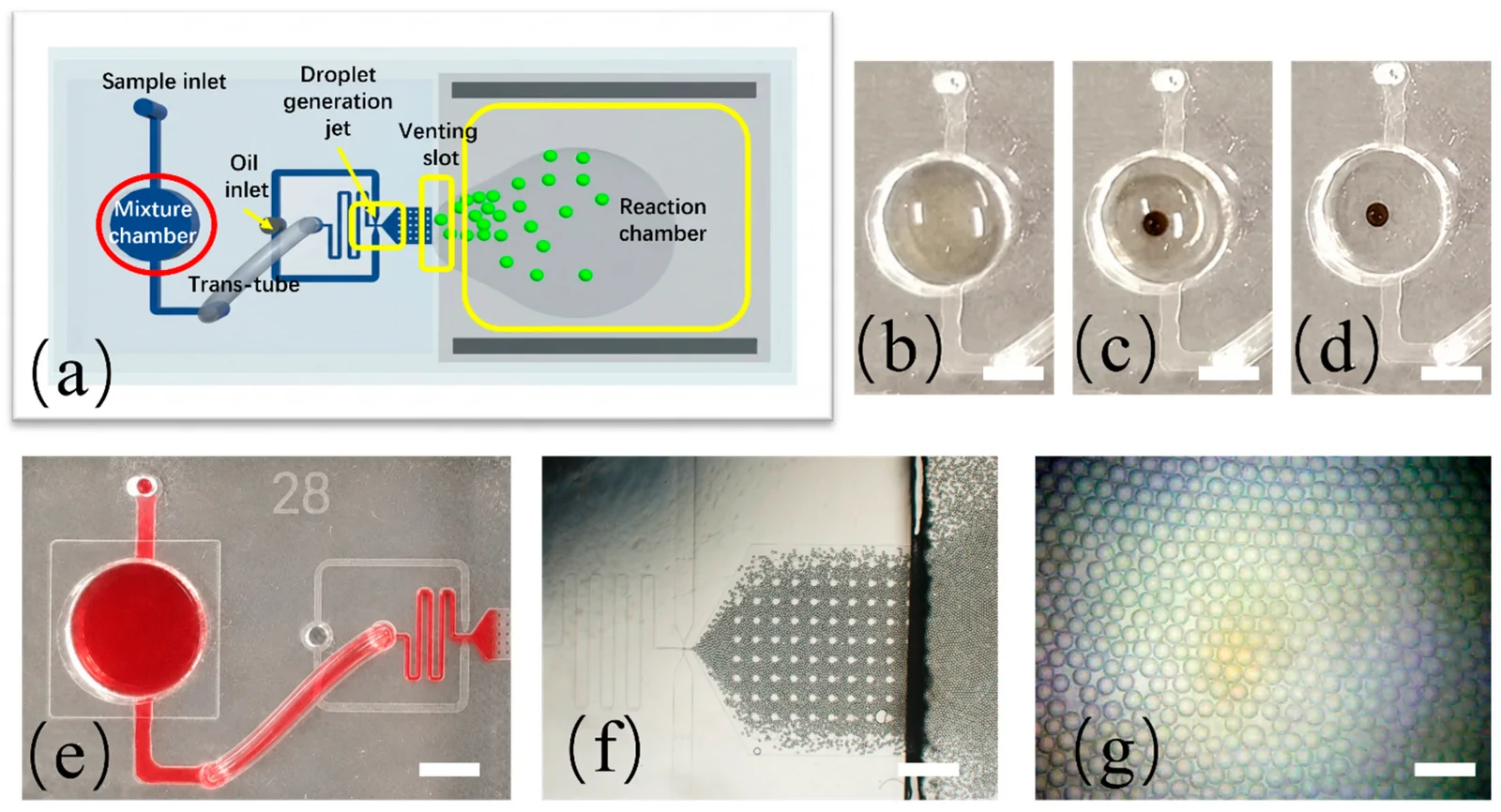
Integrated microfluidic device and representative operation processing. (**a**) Device layout showing the sample inlet, processing chamber, oil inlet, transfer tube, droplet-generation junction, venting region and reaction chamber. (**b**–**d**) Distribution and magnetic concentration of beads in the processing chamber; scale bar: 0.5 cm. (**e**) Dye-filled chip showing fluidic continuity between the processing and droplet-generation modules; scale bar: 1 cm. (**f**) Droplet accumulation in the reaction chamber; scale bar: 0.4 cm. (**g**) High-throughput, uniformly sized droplets generated by the on-chip flow-focusing; scale bar: 200 µm.

The microfluidic chip integrates four principal functional components: a sample-processing chamber, transfer tubing, a flow-focusing droplet generator, and a reaction chamber incorporating a venting region (Figure 2a). Sequential images illustrate the dispersion and magnetic concentration of the immunomagnetic beads within the processing chamber under an externally applied magnetic field (Figure 2b–d). A dye-injection experiment further demonstrated fluidic continuity between the processing chamber and the downstream droplet-generation module (Figure 2e). Upon entering the reaction chamber, the generated droplets assembled into a densely packed single-layer array (Figure 2f). Microscopic observation further showed the continuous generation of uniformly sized droplets at the flow-focusing junction (Figure 2g). Together, these observations demonstrate stable fluid transfer, magnetic manipulation, droplet generation, and array formation within the integrated device.

### 3.3. Characterization of EVs and Immunomagnetic Beads

**Figure 3:**
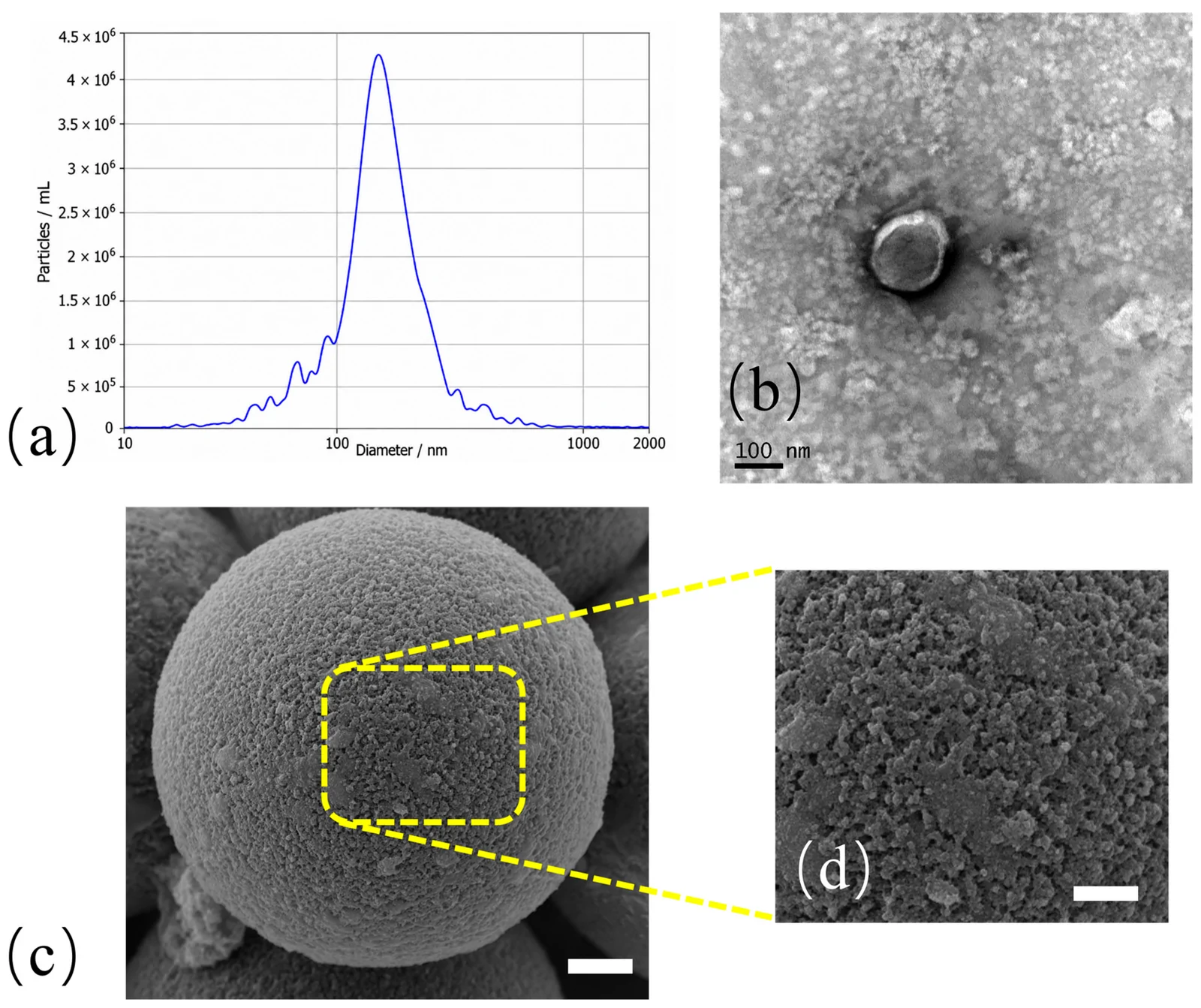
Characterization of extracellular vesicles and immunomagnetic beads. (**a**) Nanoparticle tracking analysis showing the particle-size distribution of the isolated EV preparation. (**b**) Transmission electron micrograph of a representative nanoscale vesicle. (**c**,**d**) Scanning electron micrographs of an immunomagnetic bead and its surface morphology at higher magnification; scale bar: 1 µm (**c**) and 0.5 µm (**d**).

The characterization demonstrates the size and morphological features of extracellular vesicles (Figure 3). Nanoparticle tracking analysis (NTA) revealed a prominent nanoscale particle distribution centered within the typical EV range (~120 nm) (Figure 3a). Transmission electron microscopy (TEM) further confirmed the classic cup- or saucer-shaped membrane-bound vesicle morphology (Figure 3b). Additionally, scanning electron microscopy (SEM) characterization depicted the spherical geometry and nanoscale rough surface topography of the anti-CD63 functionalized immunomagnetic beads at lower and higher magnifications (Figure 3c,d). These combined morphological observations validate the quality of the prepared EV samples and functionalized bead materials used for on-chip magnetic capture.

### 3.4. Quantitative Response and Automated Droplet Analysis

**Figure 4:**
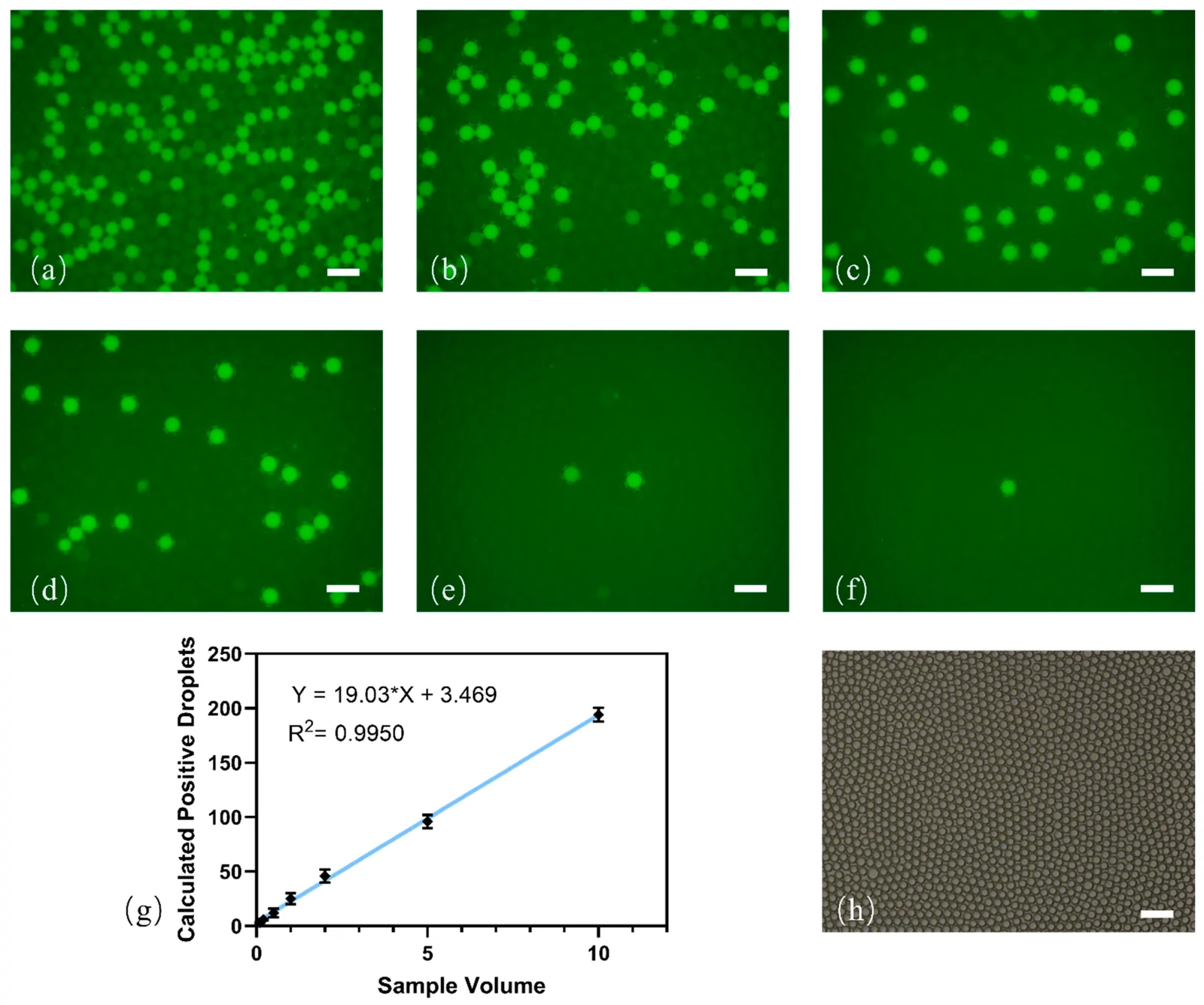
Input-dependent fluorescence response and droplet-array stability. (**a**–**f**) Representative fluorescence micrographs obtained from 10, 5, 2, 1, 0.5 and 0.2 μL of original sample, respectively, scale bar: 100 µm. Buffer was added to maintain the same final reaction volume for all conditions. (**g**) Linear relationship between analytical output and original-sample input volume across the tested range (R^2^ = 0.9950). Data are mean ± SD from three replicate measurements. (**h**) Bright-field micrograph acquired after on-chip PCR thermal cycling, showing dense droplet packing and uniform morphology; scale bar: 100 µm.

To evaluate the quantitative accuracy and dynamic response of the integrated system across broad sample concentrations, gradient input experiments were performed using 10, 5, 2, 1, 0.5, and 0.2 µL of original EV samples, with total reaction volumes equalized by buffer addition. Representative fluorescence micrographs revealed a clear, progressive decrease in positive droplet counts corresponding to decreasing sample inputs (Figure 4a–f). Using GAPDH as an internal reference gene, the calculated target copy numbers—corrected by Poisson distribution statistics—demonstrated remarkable linearity across a 50-fold input range (R^2^ = 0.9950, *n* = 3) (Figure 4g). Bright-field micrographs acquired post-PCR thermal cycling confirmed that the arrayed droplets maintained dense packing, structural integrity, and uniform morphology without coalescing (Figure 4h), validating the stability of the arrayed droplets during thermal cycling.

**Figure 5:**
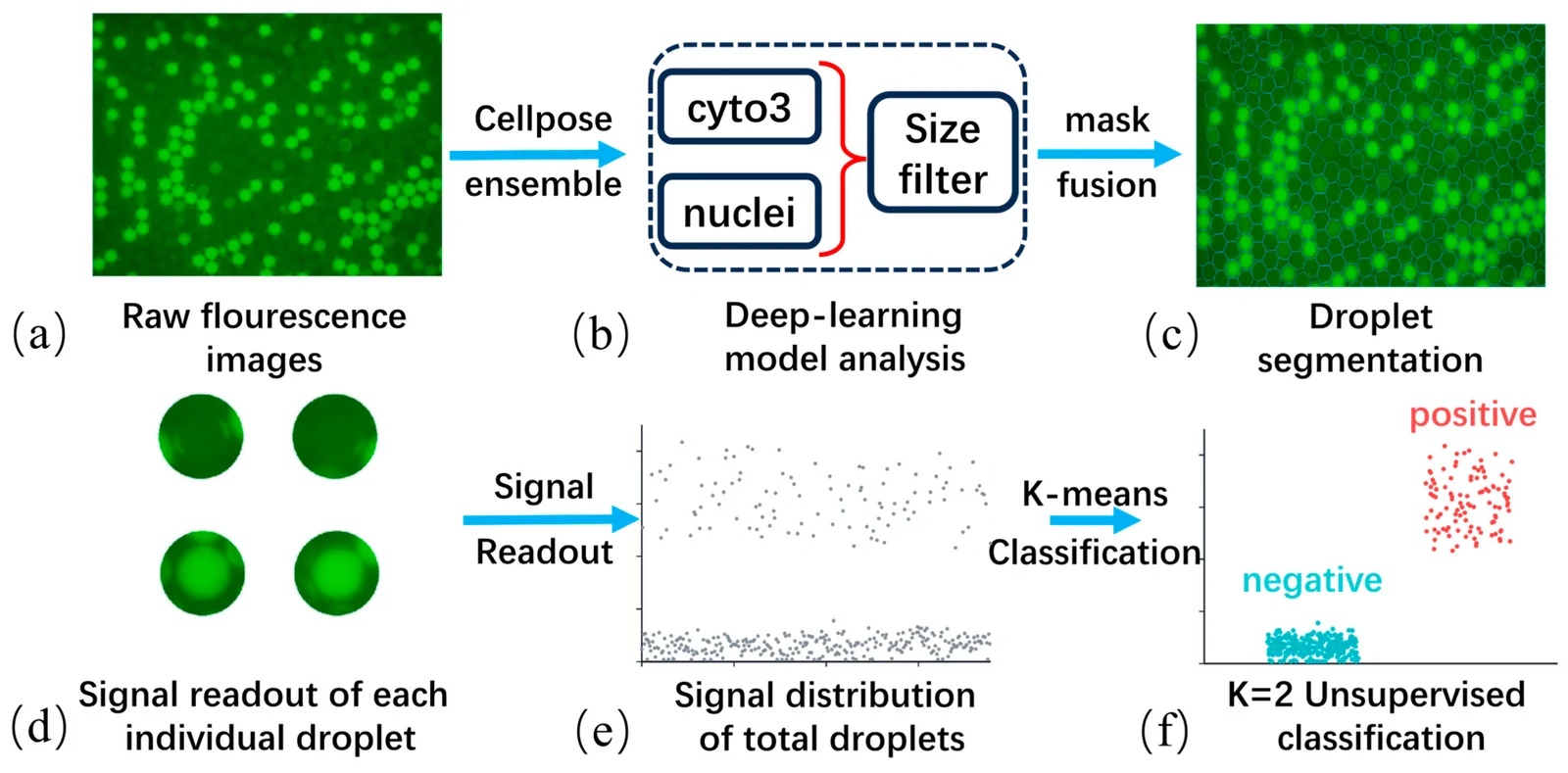
Deep-learning-assisted workflow for droplet segmentation and fluorescence classification. (**a**) Raw fluorescence micrograph. (**b**) Image representations and pretrained models used for droplet recognition. (**c**) Cellpose segmentation overlay showing the boundaries of identified droplets. (**d**) Representative droplets with low and high fluorescence. (**e**) Background-corrected signals extracted from individual indexed droplets. (**f**) Unsupervised K-means classification of eligible droplets into negative and positive populations.

To achieve objective fluorescence interpretation, a deep-learning-assisted image-processing pipeline was implemented to convert fluorescence micrographs into droplet-resolved analytical outputs through sequential image processing and classification steps (Figure 5). Cellpose-based instance segmentation was first applied to identify individual droplets and generate corresponding droplet masks (Figure 5a–c). Each detected droplet was individually indexed, while its spatial position and morphological information were retained for subsequent fluorescence quantification. Representative low- and high-intensity droplets illustrate the fluorescence differences observed within the detected reaction units (Figure 5d).

Fluorescence intensity was extracted from the original image for each segmented droplet, followed by local background correction using the surrounding droplet region. The resulting background-corrected fluorescence signals were subsequently classified using unsupervised K-means clustering with k = 2 (Figure 5e,f), The lower-intensity cluster was assigned as the negative population and the higher-intensity cluster as the positive population. This workflow enables automated droplet segmentation, fluorescence quantification, and binary classification for fluorescence images without requiring a manually defined intensity threshold.

### 3.5. Parallel Detection of RNA Mutations in Cell-Line-Derived EVs

**Figure 6:**
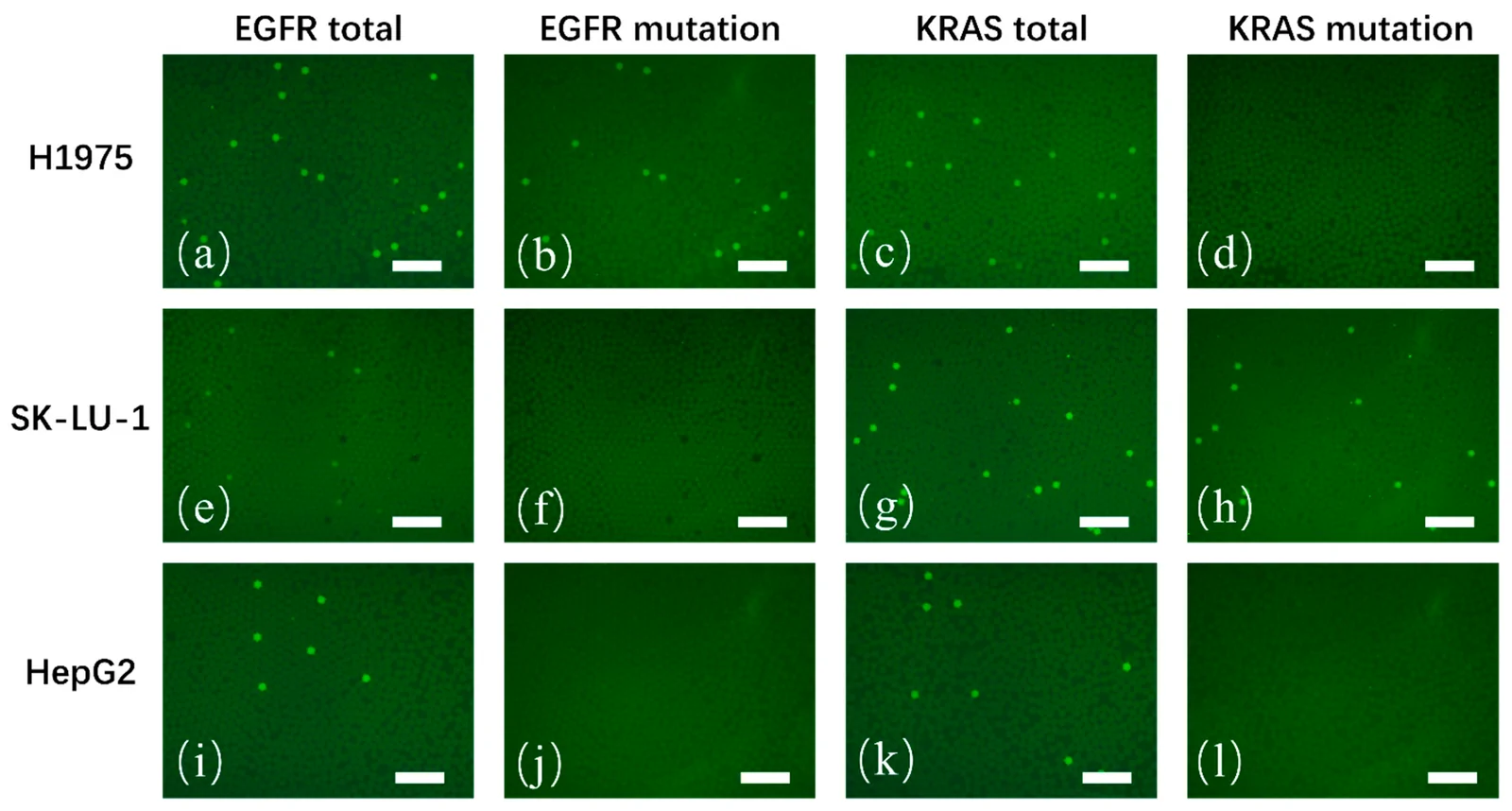
Spatially corresponding detection of EGFR L858R- and KRAS-mutation-associated signals. (**a**–**d**) NCI-H1975-derived EVs: EGFR total-target (**a**), EGFR L858R mutation-specific (**b**), KRAS total-target (**c**), and KRAS mutation-specific (**d**) fluorescence images. (**e**–**h**) Corresponding fluorescence images for SK-LU-1-derived EVs. (**i**–**l**) Corresponding fluorescence images for HepG2-derived EVs; scale bars: 100 µm. For each target assay, FAM and HEX images were sequentially acquired from the same stationary droplet field and analyzed. Because the positions of the droplets remained unchanged during imaging, total-target and mutation-specific signals could be compared at corresponding physical droplet locations. NCI-H1975-derived EVs showed an EGFR L858R-associated population, SK-LU-1-derived EVs showed a KRAS-mutation-associated population, and HepG2-derived EVs showed no discernible mutation-specific population for either target. The observed patterns were reproduced in three independent chip runs.

To evaluate the capability of the integrated platform for differential mutation profiling, EVs derived from NCI-H1975, SK-LU-1, and HepG2 cells were subjected to parallel detection of EGFR L858R and KRAS mutations (Figure 6). For each target assay, FAM and HEX fluorescence images were sequentially acquired from the same stationary droplet array and analyzed using the image-processing procedure described above. Because the droplet array remained stationary during image acquisition, the two fluorescence channels provided spatially corresponding views of the same physical reaction units, allowing total-target and mutation-specific signals to be compared at corresponding droplet positions.

NCI-H1975-derived EVs exhibited a distinct EGFR L858R mutation-associated population together with total EGFR target signals, whereas no discernible KRAS mutation-specific population was observed (Figure 6a–d). In contrast, SK-LU-1-derived EVs showed a detectable KRAS mutation-associated population but no evident EGFR L858R mutation-specific population (Figure 6e–h). HepG2-derived EVs exhibited detectable total EGFR and KRAS target signals, while no discernible mutation-specific population was observed for either target (Figure 6i–l). These mutation patterns were reproducible across three independent chip runs, demonstrating the capability of the platform to distinguish different EV RNA mutation profiles among the investigated cell-line-derived samples.

## 4. Discussion

In this study, we demonstrated a deep-learning-assisted integrated microfluidic platform combining EV processing, droplet RT-ddPCR, and automated fluorescence image analysis within a coordinated analytical workflow. By integrating EV isolation, lysis, reaction preparation, droplet generation, thermal amplification, and subsequent image-based signal analysis, the system reduces the number of manual transfer steps and the associated risks of sample loss and cross-contamination. The platform therefore combines integrated microfluidic processing with automated droplet-level image interpretation for EV RNA mutation analysis.

The microfluidic architecture provides the physical capacity and operational stability required for high-throughput digital analysis. The large-volume processing chamber accommodates increased sample input. Coupled with the flow-focusing droplet generator, the system produces up to approximately 100,000 monodisperse picoliter reaction droplets per experiment. The reaction chamber further maintains the droplets as a dense single-layer array during thermal cycling, providing a stable configuration for subsequent fluorescence imaging. Complementing the microfluidic platform, the deep-learning-assisted image-analysis workflow combines Cellpose-based droplet segmentation with unsupervised K-means classification. This strategy enables automated identification, fluorescence quantification, background correction, and positive/negative classification of individual droplets without relying on manually defined intensity thresholds. In addition, the spatial information retained for individual droplets facilitates correspondence between fluorescence signals obtained from the same droplet array, thereby supporting droplet-level interpretation of multiplexed fluorescence results. Compared with conventional readout based on manual droplet counting or a fixed fluorescence-intensity threshold, the combined recognition and self-clustering strategy reduces operator-dependent interpretation and classifies each image according to its own signal distribution. The expected droplet scale and analysis settings can be adjusted while retaining the same analytical logic, supporting extension to images with different droplet sizes and fluorescence channels. This adaptability provides a practical basis for broader EV RNA mutation profiling and future validation with clinical biofluid samples.

The analytical performance of the platform was further evaluated through input-dependent gradient experiments and mutation profiling of cell-line-derived EVs. A linear relationship was observed between original-sample input and the calculated analytical output over a wide border range (R^2^ = 0.9950), supporting the quantitative response of the integrated system under the tested conditions. Furthermore, NCI-H1975-, SK-LU-1-, and HepG2-derived EVs exhibited distinguishable EGFR L858R and KRAS mutation profiles. The paired readout is particularly important for interpreting low-abundance mutation signals in partitioned EV RNA samples. A positive total-target channel establishes the presence of amplifiable EGFR or KRAS RNA within a reaction unit, whereas the mutation-specific channel identifies the sequence-defined subset of those target-positive droplets. Because the two fluorescence fields represent the same stationary array, mutation-associated events can be assessed at spatially corresponding droplet positions rather than inferred from separately sampled populations. Mutation-specific positive droplets are therefore expected to occur within the corresponding total-target-positive population, while not every total-target-positive droplet is expected to carry the mutation-associated signal. This coordinate-preserved comparison provides a second level of verification at the individual-droplet level and reduces the likelihood that isolated bright spots, dye aggregates, or other channel-specific artifacts are interpreted as mutation-positive events. In this way, digital partitioning supports the resolution of rare targets, while the paired fluorescence readout strengthens the traceability of mutation interpretation in heterogeneous EV-derived RNA samples.

While the present study successfully demonstrated the system’s capability for EV nucleic acid analysis using cell-line-derived EVs, Western blot characterization of the EV preparations and evaluation with more complex clinical samples, such as patient plasma, were not performed. Future work will therefore focus on further validating the analytical utilities using complementary methods and adapting the workflow for direct analysis of clinical biofluid samples.

## 5. Conclusions

In this work, we developed a deep-learning-assisted integrated microfluidic platform for automated EV RNA mutation analysis. The system integrates EV isolation and lysis, droplet RT-ddPCR, thermal amplification, fluorescence imaging, and automated droplet-level analysis within a coordinated analytical workflow, enabling high-throughput droplet-level quantification with reduced manual intervention. The integration of microfluidic processing with Cellpose-based segmentation and K-means unsupervised fluorescence classification further enables automated identification and quantification of fluorescence signals without relying on manually defined thresholds. The stable droplet-array configuration and droplet-resolved fluorescence analysis support spatially traceable interpretation of multiplexed signals.

The platform was successfully applied to cell-line-derived EVs for the analysis of EGFR L858R and KRAS mutations, revealing distinguishable mutation profiles among different biological sources. These results demonstrate the feasibility of combining integrated EV processing, digital nucleic acid amplification, and automated image analysis within a unified analytical platform. In summary, this integrated microfluidic system provides a practical and automated approach for EV nucleic acid profiling and holds potential for liquid biopsy applications.

## Data Availability

The source code and model-inference workflow used for droplet segmentation, fluorescence quantification, K-means classification and result export are publicly available at https://github.com/stardust202501/droplet-analysis/tree/main/source, accessed on 24 July 2026.

## Supplementary Materials

The following supporting information can be downloaded at https://www.mdpi.com/article/10.3390/mi17101193/s1, Supplementary Method S1: Microfluidic geometry and pressure-controlled droplet generation; Supplementary Method S2: Deep-learning-assisted droplet recognition and fluorescence classification; Supplementary Method S3: Computational environment; Supplementary Method S4: Code availability.
