# Supplementary material for: Deep-Learning-Assisted Integrated Microfluidic Platform for Automated Analysis of EV RNA Mutations
Source: Micromachines (Basel). 2026 Oct 15;17(10):1193. doi: 10.3390/mi17101193 (PMC13707912; doi:10.3390/mi17101193)
Supplement: Supplementary file 1 [file micromachines-17-01193-s001.zip › micromachines-4585830-supplementary.pdf]

Supplementary Methods

Deep-learning-assisted integrated microfluidic platform for automated analysis of EV RNA mutations

S1. Microfluidic geometry and pressure-controlled droplet generation

The integrated device comprised a cylindrical EV-processing chamber, a transfer microtube, a cross-shaped flow-focusing droplet generator, a venting region and a glass reaction chamber. The processing chamber was 8 mm in diameter and 5 mm in height, providing a processing capacity greater than 120 μL. The droplet reaction chamber was assembled from two glass slides separated by a 50-μm-thick double-sided adhesive spacer, allowing the generated droplets to be retained as a densely packed single-layer array.

The continuous phase was mineral oil containing 3% (w/w) Abil EM 90 and 0.1% (w/w) Triton X-100. This formulation was used throughout the droplet-generation experiments.

S2. Deep-learning-assisted droplet recognition and fluorescence classification

Fluorescence micrographs were processed using the pretrained Cellpose cyto3 and nuclei instance-segmentation models in inference mode. The analysis was tailored to densely packed microscopic droplet fields and directly processed the experimental images under the same documented parameter set. Unlike a task-specific supervised model constructed from newly annotated images, this inference architecture does not require division of the experimental images into training, validation and test subsets; accordingly, training/validation/test percentages and a supervised average-accuracy metric are not applicable. The expected droplet diameter was set to 40 pixels for the present images and can be adjusted from 20 to 80 pixels to accommodate different apparent droplet sizes and acquisition magnifications. The outputs comprise non-rectangular instance contours, centroid coordinates and overlay images, providing spatial information beyond rectangular detection regions.

The cyto3 model was applied to the source image with a flow threshold of 0.4 and a cell-probability threshold of −1.0. The nuclei model was applied to locally enhanced and halo-suppressed representations with a flow threshold of 1.4 and a cell-probability threshold of 0.5. Candidate masks were retained between the larger of 120 pixels or 10% of the expected droplet area and 2.5 times the expected droplet area. Duplicate detections were removed using a centre-distance threshold of 0.42 times the expected diameter, and masks with more than 20% overlap with an accepted mask were excluded. Droplets touching image boundaries were recorded separately and

excluded from fluorescence clustering and final counts to avoid partial-object measurements. Image enhancement supported droplet recognition only; all fluorescence values were extracted from the original-resolution source image.

For each accepted droplet mask, fluorescence intensity was corrected using the local background surrounding the droplet. The resulting droplet-level signals were separated into negative and positive groups by unsupervised K-means clustering (k = 2; random_state = 42; n_init = 20). This self-clustering procedure does not require a manually fixed fluorescence-intensity threshold. The same analysis sequence can be applied independently to different fluorescence channels, while the expected droplet scale and recognition parameters can be adjusted for different image-acquisition conditions.

S3. Computational environment

The analysis was performed on a Windows computer equipped with an AMD Ryzen 7 7735HS processor (8 physical cores and 16 logical processors) and 24 GB RAM. Cellpose and PyTorch inference was performed on the CPU (gpu=False) without dedicated GPU acceleration. The software environment comprised Python 3.9.23, Cellpose 3.1.1.1, PyTorch 2.6.0, OpenCV 4.11.0 and scikit-learn 1.6.1. The CPU-only configuration enables the workflow to be run on a standard personal computer without specialized GPU hardware.

S4. Code availability

The source code used for droplet recognition, fluorescence extraction and unsupervised classification is available at https://github.com/stardust202501/droplet-analysis/tree/main/source.
